# Sex differences in gene regulation in the dorsal root ganglion after nerve injury

**DOI:** 10.1186/s12864-019-5512-9

**Published:** 2019-02-19

**Authors:** Kimberly E. Stephens, Weiqiang Zhou, Zhicheng Ji, Zhiyong Chen, Shaoqiu He, Hongkai Ji, Yun Guan, Sean D. Taverna

**Affiliations:** 1 Department of Pharmacology and Molecular Sciences, School of Medicine, Center for Epigenetics, Johns Hopkins University, Baltimore, MD USA; 2 Department of Biostatics, Bloomberg School of Public Health, Johns Hopkins University, Baltimore, MD USA; 3 Department of Anesthesia and Critical Care Medicine, School of Medicine, Johns Hopkins University, Baltimore, MD USA

**Keywords:** Dorsal root ganglion, Sex differences, RNA-seq, Gene expression, Peripheral nervous system, Nerve injury

## Abstract

**Background:**

Pain is a subjective experience derived from complex interactions among biological, environmental, and psychosocial pathways. Sex differences in pain sensitivity and chronic pain prevalence are well established. However, the molecular basis underlying these sex dimorphisms are poorly understood particularly with regard to the role of the peripheral nervous system. Here we sought to identify shared and distinct gene networks functioning in the peripheral nervous systems that may contribute to sex differences of pain in rats after nerve injury.

**Results:**

We performed RNA-seq on dorsal root ganglia following chronic constriction injury of the sciatic nerve in male and female rats. Analysis from paired naive and injured tissues showed that 1513 genes were differentially expressed between sexes. Genes which facilitated synaptic transmission in naïve and injured females did not show increased expression in males.

**Conclusions:**

Appreciating sex-related gene expression differences and similarities in neuropathic pain models may help to improve the translational relevance to clinical populations and efficacy of clinical trials of this major health issue.

**Electronic supplementary material:**

The online version of this article (10.1186/s12864-019-5512-9) contains supplementary material, which is available to authorized users.

## Background

Once established chronic pain is often resistant to existing treatments and associated with adverse health outcomes such as decreased quality of life [1], alterations in mood [2] and sleep patterns [3], and disability [4]. Sex differences in the susceptibility to most chronic pain conditions are well established [5] and experimental pain studies consistently demonstrate greater pain sensitivity, increased pain ratings, and decreased tolerance to a variety of pain modalities in women versus men [5–9]. However, the mechanisms underpinning these sex dimorphisms are poorly understood.

While the inclusion of women in clinical trials has increased dramatically during the past 20 years, a strong bias towards the exclusive use of male animals in preclinical studies of neuropathic pain has persisted [10, 11]. Arguably, the limited use of females in mechanistic studies of neuropathic pain has obfuscated our understanding, management, and treatment of neuropathic pain in either sex [10, 11]. The few studies that have used rodent models of chronic pain to examine sex-specific differences have primarily focused on the hormonal and neuroimmune effects on pain modulation in the central nervous system (CNS) [12]. For example, activated immune cells (e.g., microglia) in the spinal cord dorsal horn release inflammatory mediators (e.g., cytokines) in response to tissue damage which promote neuronal excitability [13]. Although the role of microglia-neuronal signaling pathways in pain pathophysiology had been characterized using predominantly male mice, recent evidence demonstrated that female and male mice use distinct immune cell types to modulate pain behaviors [14, 15].

Chronic pain following peripheral nerve injury is associated with profound changes in gene expression that alter synaptic plasticity and neuroimmune interactions. The majority of studies have examined gene expression changes exclusively in males [10, 11, 16]. One notable exception is a 2006 study by LaCroix-Fralish and colleagues [17] in which microarrays were used to measure the gene expression in the rat dorsal horn after spinal nerve ligation in both male and female rats. Despite the potential for gene expression to yield insight into the molecular underpinnings of chronic pain, a transcriptome-wide assessment of gene expression in the peripheral nervous system (PNS) after nerve injury in male and female rats has not been reported.

Here we sought to identify the similarities and differences in gene regulation between male and female rats in the PNS following peripheral nerve injury. We compare large-scale transcriptome analyses of dorsal root ganglia (DRGs) from male and female rats, in both naive and a peripheral nerve injury model, and identified differential expression of mediators of neurochemical mechanisms. Our RNA-seq suggests that data sexually dimorphic roles of sensory neurons and glia in the DRG may be an important consideration in clinical drug trials designed to evaluate treatments for chronic pain.

## Results

**Fig. 1:**
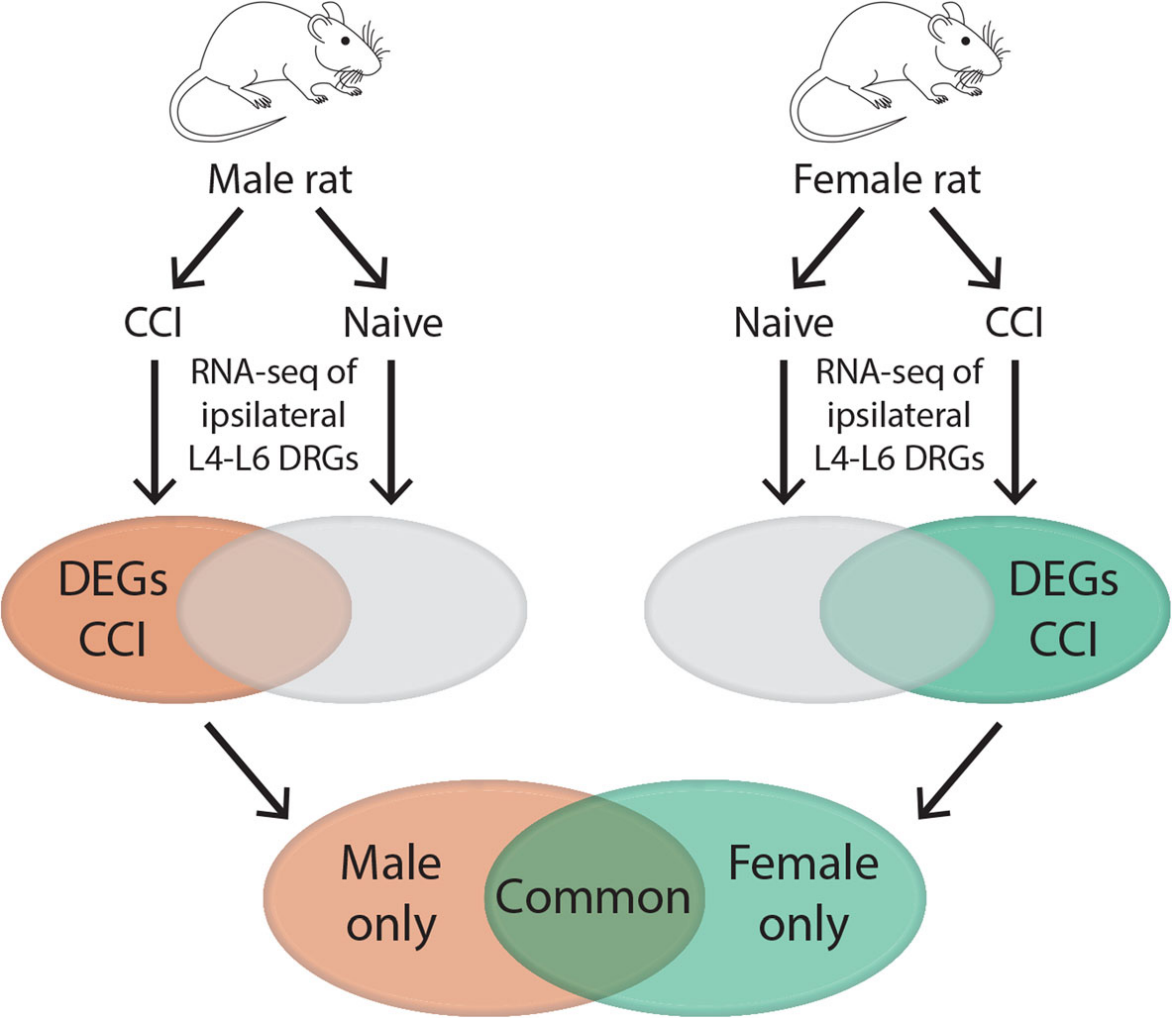
Schematic diagram of experimental procedures. Male and female rats were randomly assigned to the naïve group or receive CCI. Total RNA was isolated from the L4-L6 DRG of naïve rats and on day 14 after CCI to the sciatic nerve. Libraries were constructed after poly(A) selection and sequenced. RNA-seq was performed on ipsilateral L4-L6 DRGs from each animal. Differentially expressed genes (DEG), defined as genes expressed after CCI versus naïve with a |log_2_fold change (FC)| > 0.5 and an FDR < 0.05 were compared between male and female rats. DRG = dorsal root ganglion; CCI = chronic constriction injury. Line art of rat in this figure and herein were drawn by KES

RNA-seq transcriptome profiles of DRGs from gonadally intact, adult male and female rats were measured from naïve animals and animals at 14 days after sciatic chronic constriction injury (CCI) (Fig. 1). Basic biological influences that regulate responses to nociceptive stimuli and the generation of chronic pain have largely been investigated using neuropathic pain models in rodents. In an effort to isolate the pain behaviors from other experimental procedures used to create the model the majority of these prior studies used sham procedures as a comparison group. In sham procedures, all experimental procedures are performed with the exception of nerve injury from mechanical manipulation or administration of an active compound. In the present study, we use naïve animals as a comparison group so that we may capture all changes that are associated with clinical neuropathic pain conditions (e.g., skin incision, damage to nerve terminals, deep muscle tissue with associated inflammatory response).

### Differentially regulated genes in DRGs of naïve male and female rats

**Fig. 2:**
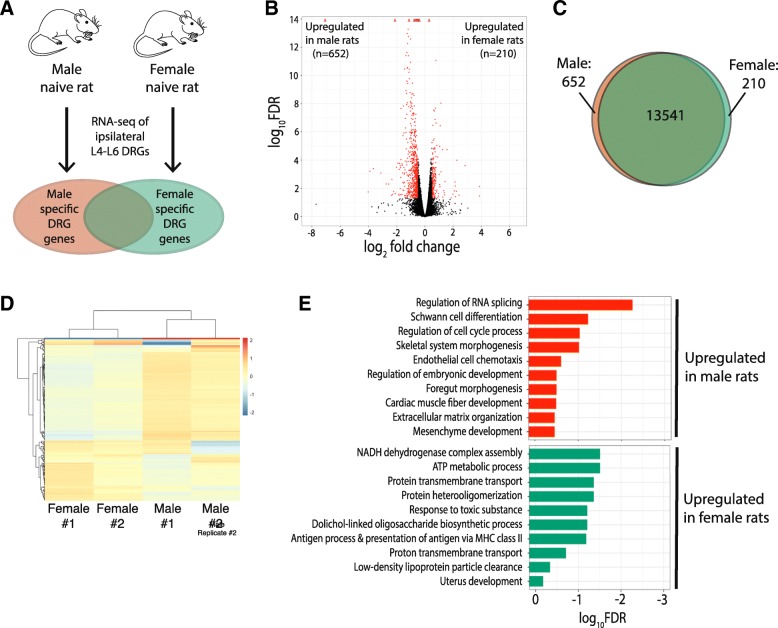
Sex differences of gene expression in DRGs from naïve rats. **a** Schematic diagram of experiment. RNA-seq performed on ipsilateral L4-L6 DRGs from naïve male and female rats. Differentially expressed genes (DEG) in one sex are identified by a |log_2_fold change (FC)| > 0.5 and an FDR < 0.05. **b** Volcano plot of differential gene expression in naïve rats between male and female rats. Significant genes are designated in red. Fold change represents the ratio of gene expression in female to male rats. **c** Venn diagram representing the number of genes expressed in males (orange), females (green), and in both sexes (overlap). **d** Heatmap that show the log_2_FC of the 200 most variable genes between naïve male and naive female rats. **e** Gene ontology pathways associated with increased expression of genes in male (top; orange) and female (bottom; green) naïve DRG. *DRG* dorsal root ganglion

To identify sex-specific differences of gene expression in DRG under physiological conditions, we performed RNA-seq and measured all poly(A)-containing transcripts expressed in the L4-L6 DRG of naïve rats (Fig. 2a). Expression levels of 14,403 genes were evaluated and the majority (13,541 genes; 94.0%) showed no significant difference in expression level between males and females (Fig. 2b). Of the 862 genes with expression levels that differed significantly between males and females, 652 (75.6%) showed increased in expression in males versus females and 210 (24.4%) showed increased expression in females versus males (Fig. 2c-d). Lists of these genes with increased and decreased expression in the DRGs between male and female rats are provided as Additional file 1 and Additional file 2. These male and female gene lists were then subject to gene ontology (GO) analysis using Metascape. GO pathways associated with genes that had relatively increased expression in the male naive DRG include Regulation of RNA splicing (GO:0043484), Schwann cell differentiation (GO:0014037), and Regulation of cell cycle process (GO:0010564) (Fig. 2e). GO pathways associated with genes that had relatively increased expression in the female naive DRG were different from that in males and include NADH dehydrogenase complex assembly (GO:0010257), ATP metabolic process (GO:0046034), and Antigen process and presentation of antigen via MHC class II (GO: 0002495).

### Differentially regulated genes in DRGs after nerve injury in male rats

**Fig. 3:**
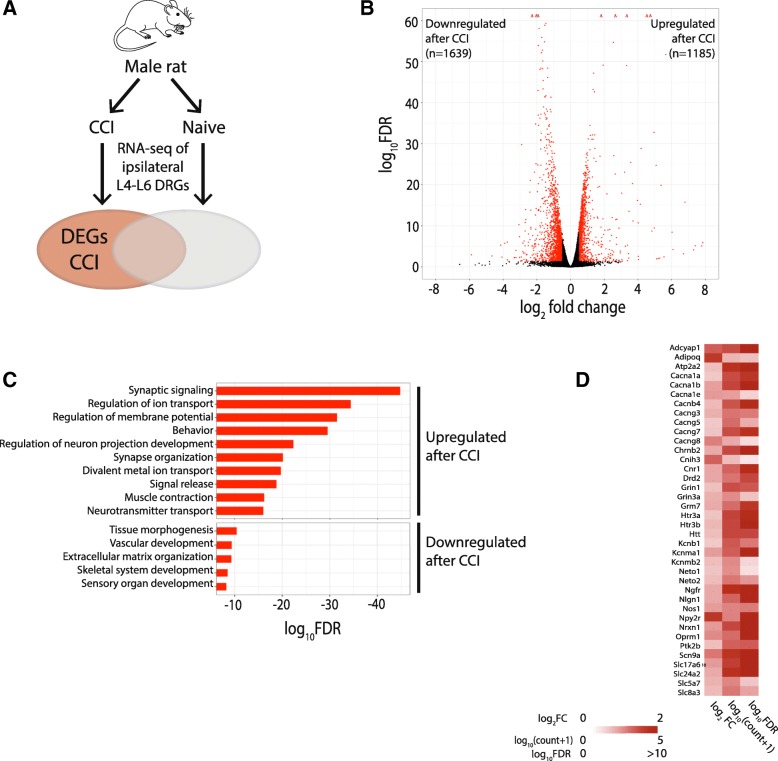
Differential gene expression between CCI and naïve groups in male rats. **a** Schematic diagram of experiment. Male rats were randomly assigned to the naïve group or receive CCI. RNA-seq performed on ipsilateral L4-L6 DRGs from each animal. Differentially expressed genes (DEG) are defined as genes expressed after CCI versus naïve with a |log_2_FC| > 0.5 and an adjusted *p*-value < 0.05. **b** Volcano plot showing RNA-seq data of DRGs from naïve male rats and male rats following CCI. DEGs are designated in red. Triangles represent genes with extremely high log_10_FDR values. **c** Gene ontology pathways associated with increased (top) and decreased (bottom) differential expression in CCI versus naive. **d** Heatmap that shows the log_2_FC, log_10_(gene count + psuedocount of 1), and the –log_10_FDR for the upregulated genes enriched in the top 3 GO terms shown in **c**). *DRG* dorsal root ganglia, *CCI* chronic constriction injury, *FC* fold change, *FDR* false discovery rate

To determine the effects of peripheral nerve injury on gene expression in the DRGs of male rats, we evaluated RNA-seq data obtained 14 days after CCI and compared these expression profiles to those from naive males (Fig. 3a). Compared to naive DRGs, DRGs from male CCI rats differentially expressed 2824 genes (19.5%) (Fig. 3b). Of these 2824 differential genes, 1185 genes (42.0%) were upregulated after injury and 1639 genes (58.0%) were downregulated after injury (Fig. 3b). Despite the use of naïve rats as the comparison group, we found that a large number of upregulated genes after CCI (e.g., *Reg3b*, *Vgf*, *Atf3*, *Cacna2d1*, *Gal*, *Npy*, *Gap43*) are the same as those previously reported to be upregulated in the DRG after nerve injury which used sham-operated rats as the comparison group [16, 18]. The top 25 upregulated and downregulated genes after CCI ranked by the false discovery rate (FDR) are listed in Table 1 and Table 2, respectively. GO analysis of the upregulated transcripts confirmed significant enrichment among nerve injury and pain related processes (Fig. 3c). A total of 38 genes were present in the three most significant GO biological processes and include voltage-gated ion channels (e.g., Cacna1a, Cacna1b, Cacna1e, Scn9a), ligand-gated ion channels (e.g., Chrnb2, Grin1, Htr3a), and G-protein coupled receptors (e.g., Cnr1, Npy2r, Oprm1) (Fig. 3d). Among the 652 genes that showed increased expression in DRGs of naïve male rats compared to female naïve rats, 549 genes (84.2%) were downregulated following CCI whereas only 7 genes (1.1%) were upregulated following CCI.

**Table 1:** Top 25 differentially upregulated genes following CCI in males

Ensembl ID	*Symbol*	Full gene name	log2 Fold Change	Standard error	FDR
ENSRNOG00000003745	*Atf3*	Activating transcription factor 3	2.59	0.11	1.10E-121
ENSRNOG00000004805	*Stac2*	SH3 and cysteine rich domain 2	4.58	0.20	3.87E-111
ENSRNOG00000006151	*Reg3b*	Regenerating family member 3 beta	4.74	0.23	1.76E-89
ENSRNOG00000010079	*Car3*	Carbonic anhydrase 3	3.32	0.17	5.73E-80
ENSRNOG00000033531	*Cacna2d1*	Calcium voltage-gated channel auxiliary subunit alpha2delta 1	1.80	0.10	1.89E-71
ENSRNOG00000015156	*Gal*	Galanin and GMAP prepropeptide	2.54	0.16	2.02E-55
ENSRNOG00000018808	*Vip*	Vasoactive intestinal peptide	5.65	0.36	1.79E-52
ENSRNOG00000010803	*Gabra5*	Gamma-aminobutyric acid type A receptor alpha 5 subunit	1.93	0.13	7.30E-50
ENSRNOG00000056493	*Mybpc1*	Myosin binding protein C, slow type	3.33	0.22	8.95E-50
ENSRNOG00000017333	*Syt4*	Synaptotagmin 4	1.37	0.09	6.47E-48
ENSRNOG00000049882	*Adcyap1*	Adenylate cyclase activating polypeptide 1	1.34	0.09	6.37E-44
ENSRNOG00000013654	*Cbln2*	Cerebellin 2 precursor	1.42	0.10	2.33E-43
ENSRNOG00000006639	*Scn9a*	Sodium voltage-gated channel alpha subunit 9	1.16	0.09	3.63E-35
ENSRNOG00000010478	*Serpina3n*	Serine (or cysteine) peptidase inhibitor, clade A, member 3 N	4.95	0.40	1.71E-33
ENSRNOG00000015220	*Crtac1*	Cartilage acidic protein 1	1.43	0.12	7.35E-33
ENSRNOG00000007324	*Plxna2*	Plexin A2	1.30	0.10	7.35E-33
ENSRNOG00000004874	*Flrt3*	Fibronectin leucine rich transmembrane protein 3	1.18	0.10	9.78E-33
ENSRNOG00000004067	*Nrcam*	Neuronal cell adhesion molecule	1.05	0.09	7.15E-32
ENSRNOG00000047466	*Bdnf*	Brain-derived neurotrophic factor	1.34	0.11	8.21E-32
ENSRNOG00000024832	*Gpr158*	G protein-coupled receptor 158	1.26	0.10	8.77E-32
ENSRNOG00000053334	*Stmn4*	Stathmin 4	1.45	0.12	1.46E-29
ENSRNOG00000018598	*Ankrd1*	Ankyrin repeat domain 1	4.19	0.36	1.47E-29
ENSRNOG00000007354	*Trpa1*	Transient receptor potential cation channel, subfamily A, member 1	1.15	0.10	3.17E-29
ENSRNOG00000017445	*Tubb2b*	Tubulin, beta 2B class IIb	1.00	0.09	1.83E-28
ENSRNOG00000011696	*Lifr*	Leukemia inhibitory factor receptor alpha	0.93	0.08	3.32E-28

**Table 2:** Top 25 differentially downregulated genes following CCI in males

Ensembl ID	*Symbol*	Full gene name	log2 Fold Change	Standard error	FDR
ENSRNOG00000016516	*Mbp*	Myelin basic protein	−2.03	0.09	1.20E-104
ENSRNOG00000018642	*Leng8*	Leukocyte receptor cluster member 8	−2.32	0.12	2.45E-80
ENSRNOG00000012906	*Bcas1*	Breast carcinoma amplified sequence 1	−1.93	0.10	3.11E-74
ENSRNOG00000020942	*Plekha4*	Pleckstrin homology domain containing A4	−1.46	0.09	4.65E-60
ENSRNOG00000026704	*Drp2*	Dystrophin related protein 2	−1.53	0.09	1.37E-59
ENSRNOG00000010626	*Sphk1*	Sphingosine kinase 1	−1.86	0.11	8.00E-59
ENSRNOG00000025768	*Clk1*	CDC-like kinase 1	−1.71	0.10	1.23E-56
ENSRNOG00000018369	*Prx*	Periaxin	−1.54	0.10	1.45E-55
ENSRNOG00000013387	*Tpcn2*	Two pore segment channel 2	−1.91	0.12	1.16E-54
ENSRNOG00000030266	*Plekhg2*	Pleckstrin homology and RhoGEF domain containing G2	−1.67	0.11	1.58E-53
ENSRNOG00000008782	*Pnisr*	PNN interacting serine and arginine rich protein	−1.72	0.11	6.57E-53
ENSRNOG00000048139	*Ncmap*	Noncompact myelin associated protein	−1.65	0.11	7.10E-51
ENSRNOG00000061731	*Plxnb3*	Plexin B3	−1.76	0.12	3.68E-49
ENSRNOG00000003809	*Sat1*	Spermidine/spermine N1-acetyl transferase 1	−1.36	0.09	3.78E-49
ENSRNOG00000020763	*Snrnp70*	Small nuclear ribonucleoprotein U1 subunit 70	−1.66	0.11	5.41E-49
ENSRNOG00000017309	*Rsrp1*	Arginine and serine rich protein 1	−1.81	0.12	9.01E-49
ENSRNOG00000060614	*Pxdn*	Peroxidasin homolog (Drosophila)	−1.35	0.09	7.10E-47
ENSRNOG00000018748	*Slc16a11*	Solute carrier family 16, member 11	−1.62	0.11	3.90E-46
ENSRNOG00000020525	*Col5a3*	Collagen type V alpha 3 chain	−1.63	0.11	4.46E-44
ENSRNOG00000007657	*Col27a1*	Collagen type XXVII alpha 1 chain	−1.93	0.13	1.02E-43
ENSRNOG00000029535	*Nrbp2*	Nuclear receptor binding protein 2	−1.46	0.10	2.00E-42
ENSRNOG00000009951	*Aif1l*	Allograft inflammatory factor 1-like	−1.52	0.11	3.17E-42
ENSRNOG00000011921	*Dusp4*	Dual specificity phosphatase 4	−1.52	0.11	4.30E-42
ENSRNOG00000001254	*Col6a2*	Collagen type VI alpha 2 chain	−1.16	0.08	4.72E-42
ENSRNOG00000006209	*Mbd6*	Methyl-CpG binding domain protein 6	−1.60	0.12	1.12E-39

### Differentially regulated genes in DRGs after nerve injury in female rats

**Fig. 4:**
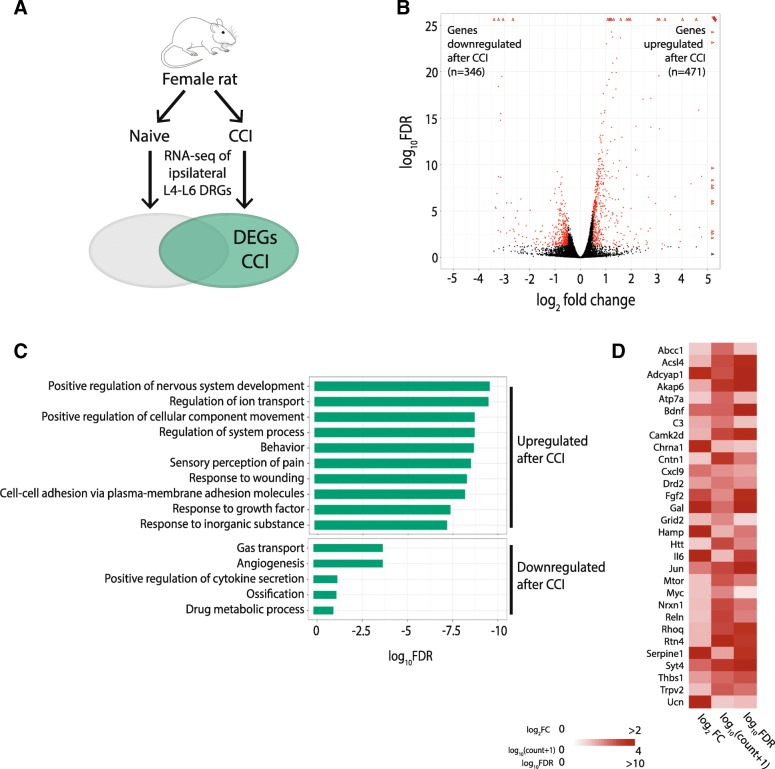
Differential gene expression between CCI and naïve groups in female rats. **a** Schematic diagram of experiment. Female rats were randomly assigned to the naïve group or receive CCI. RNA-seq performed on ipsilateral L4-L6 DRGs from each animal. Differentially expressed genes (DEG) are defined as genes expressed after CCI versus naïve with a |log_2_FC| > 0.5 and an FDR < 0.05. **b** Volcano plot showing RNA-seq data of DRGs from naïve female rats and male rats following CCI. DEGs are designated in red. Triangles represent genes with extremely high log_10_FDR and/or log_2_FC values. **c** Gene ontology pathways associated with increased (top) and decreased (bottom) differential expression in CCI versus naïve. **d** Heatmap that shows the log_2_FC, log_10_(gene count + psuedocount of 1), and the –log_10_FDR for the upregulated genes enriched in the top 2 GO terms shown in **c**). *DRG* dorsal root ganglia, *CCI* chronic constriction injury, *FC* fold change, *FDR* false discovery rate

To determine the effects of peripheral nerve injury on gene expression in female rats, we evaluated RNA-seq data at 14 days after CCI, and compared the expression profiles to those from the naive females (Fig. 4a). In comparison to naive DRGs, 817 (5.7%) genes were differentially regulated after nerve injury (Fig. 4b). Of these 817 differential genes, 471 genes (57.6%) were upregulated after injury and 346 genes (42.3%) were downregulated. The top 25 upregulated and downregulated genes after CCI ranked by DR are listed in Table 3 and Table 4, respectively. GO analysis identified that 2 of the top enriched GO biological processes in the differentially expressed genes in females are shared between males and females following nerve injury (i.e., Behavior (GO:0007610), Regulation of Ion Transport (GO:0043269); Fig. 4c). However, even though males and females share enrichment in these biological processes, just 20 and 23% of these genes are common to both males and females (Additional file 3). A total of 130 genes show enrichment in the “Positive regulation of nervous system development” GO biological process and include genes that encode for cytokines (e.g., Il6, Il1r1), chemokines (e.g., Cxcl13, Cxcl9), and enzymes (e.g., Doux2, Gch1, Tnik) (Fig. 4d). Among the 210 genes with increased expression in naive DRGs of female rats compared to the naïve male rats, 30 (14.2%) were downregulated and 4 (1.9%) were upregulated following CCI.

**Table 3:** Top 25 differentially upregulated genes following CCI in females

Ensembl ID	*Symbol*	Full gene name	log2 Fold Change	Standard error	FDR
ENSRNOG00000003745	*Atf3*	Activating transcription factor 3	3.38	0.12	5.16E-171
ENSRNOG00000004805	*Stac2*	SH3 and cysteine rich domain 2	5.12	0.20	2.98E-135
ENSRNOG00000006151	*Reg3b*	Regenerating family member 3 beta	5.91	0.25	2.92E-122
ENSRNOG00000018808	*Vip*	Vasoactive intestinal peptide	5.63	0.26	8.71E-98
ENSRNOG00000033531	*Cacna2d1*	Calcium voltage-gated channel auxiliary subunit alpha2delta 1	1.93	0.10	1.21E-74
ENSRNOG00000015156	*Gal*	Galanin and GMAP prepropeptide	3.10	0.17	1.42E-67
ENSRNOG00000001416	*Vgf*	VGF nerve growth factor inducible	1.98	0.12	1.87E-58
ENSRNOG00000049882	*Adcyap1*	Adenylate cyclase activating polypeptide 1	1.87	0.12	2.61E-51
ENSRNOG00000020136	*Tgm1*	Transglutaminase 1	5.11	0.34	4.71E-47
ENSRNOG00000004874	*Flrt3*	Fibronectin leucine rich transmembrane protein 3	1.66	0.11	1.82E-44
ENSRNOG00000010478	*Serpina3n*	Serine (or cysteine) peptidase inhibitor, clade A, member 3 N	8.15	0.60	2.29E-39
ENSRNOG00000017333	*Syt4*	Synaptotagmin 4	1.26	0.09	3.28E-37
ENSRNOG00000000065	*Pde6b*	Phosphodiesterase 6B	4.01	0.31	1.22E-34
ENSRNOG00000003031	*Atp2b4*	ATPase plasma membrane Ca2+ transporting 4	1.36	0.11	3.05E-33
ENSRNOG00000018598	*Ankrd1*	Ankyrin repeat domain 1	4.56	0.37	8.96E-33
ENSRNOG00000019447	*Ecel1*	Endothelin converting enzyme-like 1	3.05	0.25	6.10E-32
ENSRNOG00000001476	*Cldn4*	Claudin 4	6.36	0.53	1.11E-30
ENSRNOG00000017445	*Tubb2b*	Tubulin, beta 2B class IIb	1.16	0.10	7.43E-28
ENSRNOG00000012422	*Tnik*	TRAF2 and NCK interacting kinase	1.35	0.12	3.79E-27
ENSRNOG00000013521	*Dhfr*	Dihydrofolate reductase	1.25	0.11	1.04E-26
ENSRNOG00000005615	*Gadd45a*	Growth arrest and DNA-damage-inducible, alpha	1.22	0.11	5.32E-25
ENSRNOG00000013496	*Crisp3*	Cysteine-rich secretory protein 3	6.65	0.61	5.43E-25
ENSRNOG00000047466	*Bdnf*	Brain-derived neurotrophic factor	1.27	0.12	1.89E-24
ENSRNOG00000010803	*Gabra5*	Gamma-aminobutyric acid type A receptor alpha 5 subunit	1.58	0.15	2.36E-24
ENSRNOG00000001528	*Gap43*	Growth associated protein 43	1.02	0.10	1.01E-23

**Table 4:** Top 25 differentially downregulated genes following CCI in females

Ensembl ID	*Symbol*	Full gene name	log2 Fold Change	Standard error	FDR
ENSRNOG00000047098	*Hbb-b1*	Hemoglobin, beta adult major chain	−3.41	0.16	1.87E-100
ENSRNOG00000061299	*LOC100134871*	Beta globin minor gene	−3.22	0.15	5.47E-94
ENSRNOG00000045989	*Hba-a1*	Hemoglobin alpha, adult chain 1	−3.02	0.23	8.70E-37
ENSRNOG00000000167	*Alas2*	5′-aminolevulinate synthase 2	−2.73	0.24	2.67E-26
ENSRNOG00000020951	*Slc4a1*	Solute carrier family 4 member 1	−3.09	0.31	3.26E-20
ENSRNOG00000047321	*Hba2*	Hemoglobin, alpha 2	−3.23	0.34	3.87E-19
ENSRNOG00000058105	*Hbb*	Hemoglobin subunit beta	−3.14	0.36	3.24E-16
ENSRNOG00000029886	*Hba1*	Hemoglobin, alpha 1	−3.15	0.36	1.68E-15
ENSRNOG00000032002	*Hapln1*	Hyaluronan and proteoglycan link protein 1	−0.91	0.13	5.84E-10
ENSRNOG00000024330	*Ngp*	Neutrophilic granule protein	−3.22	0.48	1.97E-09
ENSRNOG00000000814	*Fabp7*	Fatty acid binding protein 7	−0.73	0.11	2.23E-09
ENSRNOG00000028707	*Np4*	Defensin NP-4 precursor	−3.14	0.47	2.35E-09
ENSRNOG00000018958	*Mt3*	Metallothionein 3	−0.77	0.12	9.01E-09
ENSRNOG00000048139	*Ncmap*	Noncompact myelin associated protein	−0.78	0.12	1.33E-08
ENSRNOG00000006471	*Pvalb*	Parvalbumin	−0.84	0.13	2.91E-08
ENSRNOG00000017619	*Aldh1a1*	Aldehyde dehydrogenase 1 family, member A1	−0.98	0.16	4.22E-08
ENSRNOG00000003809	*Sat1*	Spermidine/spermine N1-acetyl transferase 1	−0.74	0.12	8.26E-08
ENSRNOG00000010402	*Hspb2*	Heat shock protein family B (small) member 2	−1.01	0.17	1.31E-07
ENSRNOG00000011917	*Cd79b*	CD79b molecule	−3.28	0.55	1.36E-07
ENSRNOG00000016752	*Crispld2*	Cysteine-rich secretory protein LCCL domain containing 2	−0.73	0.12	1.98E-07
ENSRNOG00000019183	*Alox15*	Arachidonate 15-lipoxygenase	−2.34	0.41	5.15E-07
ENSRNOG00000013228	*Scrg1*	Stimulator of chondrogenesis 1	−1.18	0.21	7.18E-07
ENSRNOG00000010031	*Vtn*	Vitronectin	−0.67	0.12	7.18E-07
ENSRNOG00000010408	*Polr2k*	RNA polymerase II subunit K	−0.93	0.17	1.26E-06
ENSRNOG00000019660	*Spib*	Spi-B transcription factor	−2.73	0.49	1.35E-06

### Comparison of differentially regulated genes in DRGs after nerve injury between males and females

**Fig. 5:**
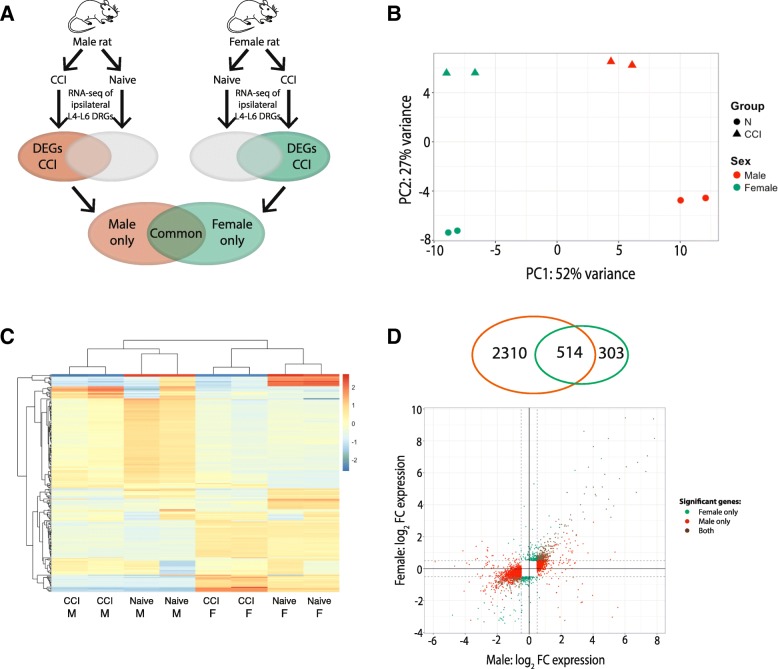
Comparison of differential gene expression between CCI and naïve groups in male and female rats. **a** Schematic diagram of experiment. Male and female rats were randomly assigned to the naïve group or receive CCI. RNA-seq performed on ipsilateral L4-L6 DRGs from each animal. Differentially expressed genes (DEG) defined as genes expressed after CCI versus naïve with a |log_2_FC| > 0.5 and an FDR < 0.05. **b** Principal component analysis of male and female rats from the naïve and CCI groups. **c** Heatmap that shows the log_2_FC of the 200 most differentially expressed genes between the CCI and Naïve groups in female and male rats. **d** Venn diagram shows the number of genes that are differentially expressed in males (orange) and females (green). Log_2_FC expression between the CCI and naive males (x-axis) and females (y-axis). Threshold of |log_2_FC| > 0.5(dashed lines) with an FDR < 0.05 designates DEGs in female rats only (green), male rats only (orange), and in both male and female rats (brown). *DRG* dorsal root ganglia, *CCI* chronic constriction injury, *FC* fold change, *FDR* false discovery rate

To identify sex-specific transcriptional changes that occur following peripheral nerve injury, we compared the lists of differentially expressed genes after CCI between male and female rats (Fig. 5a). We first visualized the extent of similarity/dissimilarity in the transcriptional profiles of the individual samples using the first two principal components from principal component analysis of all genes (Fig. 5b). The first two principal components accounted for a total of 79% of the total variance among the samples and produced distinct clusters of the samples by treatment condition (i.e., naive, CCI) and sex. This segregation of samples was confirmed by hierarchical clustering of the top 200 genes that showed the greatest variability across all groups (Fig. 5c). Of the 3127 genes that were differentially regulated between males and females, only 514 genes (16.4%) were common to both males and females regardless of direction of transcriptional changes (Fig. 5d).

**Fig. 6:**
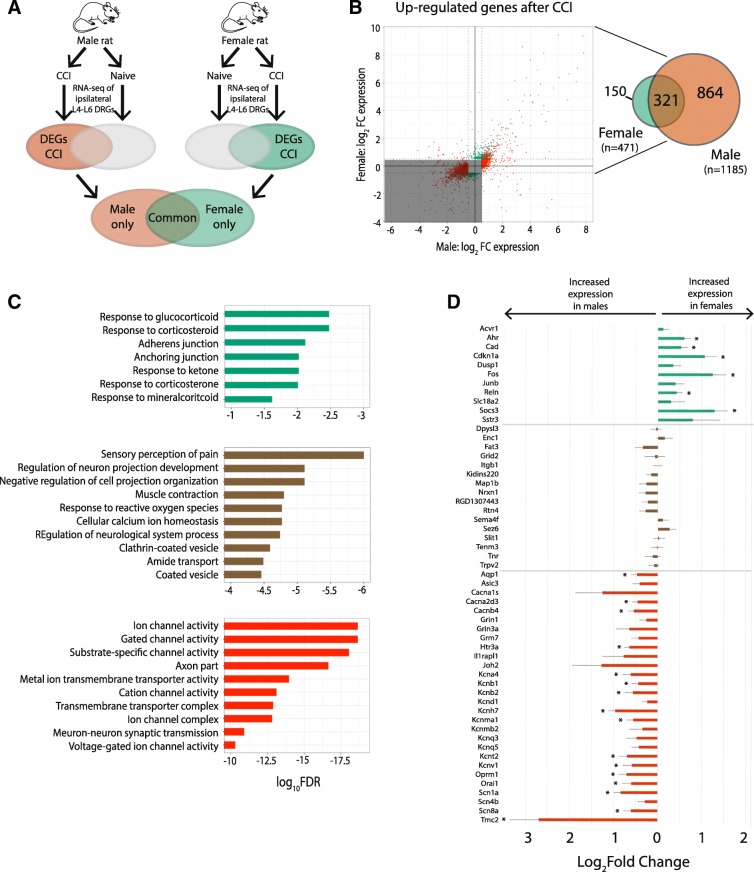
Gene ontology in differentially expressed genes that are upregulated after CCI. **a** Schematic diagram of experiment. Male and female rats were randomly assigned to the naïve group or receive CCI. RNA-seq performed on ipsilateral L4-L6 DRGs from each animal. Differentially expressed genes (DEG) defined as genes expressed after CCI versus naïve with a |log_2_FC| > 0.5 and an FDR < 0.05. **b** Log_2_FC expression between the CCI and naive males (x-axis) and females (y-axis) for DEGs upregulated in CCI versus naive. Threshold of |log_2_FC| > 0.5 (dashed lines) with an FDR < 0.05 designates DEGs in female rats only (green), male rats only (orange), and in both male and female rats (brown). Venn diagram shows the numbers of DEGs identified in each of these groups. **c** Functional pathway analysis lists the top gene ontology pathways with the FDR for each term in female rats (top; green), in both male and female rats (middle; brown) and in male rats (bottom; orange). **d** For each of the top-ranking pathways, the log_2_FC expression values for DEGs common to several of these pathways are plotted. Genes that significantly differ between males and females with an FDR < 0.05 are indicated with an asterisk. Positive values of the log_2_FC indicate increased expression in females compared to males and negative values indicate increased expression in males versus females. *DRG* dorsal root ganglia, *CCI* chronic constriction injury, *FDR* false discovery rate, *FC* fold change

We then divided all upregulated genes that had an FDR < 0.05 and |log_2_FC| > 0.5 into the following 5 groups: 1) genes upregulated in both males and females, 2) genes upregulated in females with no change of gene expression in males, 3) genes upregulated in males with no change of gene expression in females, 4) genes upregulated in females and downregulated in males, and 5) genes upregulated in males and downregulated in females (Fig. 6b). Of the 1185 genes that were upregulated in males, 321 (27.1%) were also upregulated in females. We used GeneMANIA to identify functional pathways enriched in this gene set (Fig. 6c). The top functional pathways identified in this set of upregulated genes were Sensory Perception of Pain, Regulation of Neuron Projection Development, and Negative Regulation of Cell Projection Organization.

A total of 146 genes were specifically upregulated in females after CCI and had no significant change in gene expression in males. The functional pathways identified from this set of genes include response to glucocorticoid (GO:0051384) and response to corticosteroid (GO:0051412) (Fig. 6c). Some examples of genes associated with these pathways include *Ahr*, *Cad*, *Cdkn1a*, *Fos*, *Reln*, and *Socs3* (Fig. 6d).

A total of 859 genes were specifically upregulated in males after CCI and had no expression change in females. The functional pathways identified from this set of genes include gated channel activity (GO:002836), ion cannel activity (GO:0022839), substrate-specific channel activity (GO:0022838), and metal ion transmembrane transporter activity (GO:0046873) (Fig. 6c). Examples of genes associated with these pathways include potassium channels (i.e., *Kcna4*, *Kcnb1*, *Kcnh7*, *Kcnma1*, *Kcnq5*, *Kcnt2*, *Kcnv1*), calcium channels (i.e., *Cacna2d3*, *Cacnb4*), *Oprm1*, and *Scn8a* (Fig. 6d). A complete list of genes found to be significantly differentially expressed between males and females after CCI is provided in Additional file 4. Four genes were regulated in opposite directions after injury. *Adamts4*, *Cyp2s1*, *Top2a*, and *Cenpf* were upregulated in female rats and downregulated in male rats after CCI compared with naive controls. *Actg2*, *Casq1*, *Pla2g2a*, *Acta2*, and *Clec1b* were upregulated in males and downregulated in females.

We divided the genes downregulated after CCI that had an FDR < 0.05 and |log_2_FC| > 0.5 into the following 3 groups: 1) genes downregulated in both males and females, 2) genes downregulated in females with no change of gene expression in males, 3) genes downregulated in males with no change of gene expression in females (Additional file 5B). No significant functional pathways were enriched using the genes common to both males and females or in genes significantly downregulated only in females. Genes downregulated only in females showed significant enrichment in the Mitochondrial membrane part GO cellular component (Additional file 5C). Genes downregulated only in males showed significant enrichment in pathways enriched for extracellular matrix-related pathways (Additional file 5C).

Additional file 6 shows the results of biologic validation of 10 genes by qPCR using a separate cohort of animals. We first identified an appropriate endogenous control gene by evaluating 6 candidate reference genes that showed the most stable gene expression among naïve and injured DRG of both sexes. The NormFinder algorithm calculated the lowest stability score for *Hmbs* which is consistent with stable expression among conditions (Table 5). Due to missing data from one sample *Ywhaz* could not be included in this analysis. The relative changes in gene expression derived by qPCR were in agreement with those detected by RNA-seq.

**Table 5:** Stability values of candidate reference genes in the DRG as determined by NormFinder

Ranking Order	Gene or pair of genes	Stability
1	Hmbs	0.13
2	*Hmbs* & *Actb*	0.16
3	*Hmbs* & *G6pd*	0.18
4	*Actb* & *G6pd*	0.19
5	*Actb*	0.25
6	*Sdha* & *Hmbs*	0.29
7	*G6pd*	0.30
8	*Sdha* & *Actb*	0.37
9	*Sdha* & *G6 ph*	0.38
10	*Sdha*	0.55
11	*Polr2*	0.95

## Discussion

Few studies have addressed sex-specific alterations in gene expression in neuropathic pain models, and as a result, the molecular pathways underlying sex differences in neuropathic pain are poorly understood. In addition, gene expression data are available for several types of neuronal tissue from rats, mice, and humans; however, to our knowledge, RNA-seq derived whole transcriptome analysis of the DRG, which contain the cell bodies of primary sensory neurons, has not been performed in these organisms. In the present study, we use RNA-seq to compare the gene expression profiles in lumbar DRGs between male and female rats under both naïve and nerve injury conditions. While additional biological replicates would allow additional genes with more subtle gene expression changes to be identified, our results highlight vast differences in the regulation of genes in PNS that are associated with pain-relevant pathways between females and males after injury. However, both sexes upregulate similar groups of genes associated with neuron regrowth pathways.

### Sex differences in gene expression in the naïve DRG

Our gene expression profiles from naive DRGs of male and female rats provide important insights into the normal expression of PNS genes in an unperturbed organism. Remarkably, only 6% of all genes expressed in the naïve DRGs were differentially expressed by sex. Furthermore, these genes that were differentially expressed between sexes did not enrich for pathways directly implicated in either nerve injury or nociception. However, genes that had increased expression in naïve females did show enrichment in immune-related pathways. Increased expression in females of immune-related genes is not unexpected since the connection between sex hormones and immune function is tightly linked. While comprehensive mechanisms for the sexual dimorphism in nociceptive pain have yet to be determined, current evidence supports an important role of sex hormones in pain modulation through the innate and adaptive immune systems [12, 19]. Overall, females mount a stronger humoral, cellular, and inflammatory response than males which is largely attributed to differences in circulating levels of sex hormones especially the decreased levels of androgens [20, 21]. The effects of circulating androgens are known to dampen proinflammatory cytokine production through release of IL-10 by Th1 cells [22] in males. Inflammatory cytokines modulate neuronal excitability through changes in the composition, density, and spatial distribution of ion channels and receptors in the neuronal membrane [23, 24]. Therefore, males may experience less pain as a result of a damped pro-inflammatory immune response after a pain-initiating stimulus. Indeed, evidence from experimental pain studies of pain-free individuals demonstrates a more pronounced temporal summation and greater pain sensitivity in females versus males [5].

### Gene expression that showed similar changes after nerve injury between males and females

Genes that were similarly upregulated in the DRG of both sexes after nerve injury were related to neuron regeneration, pain, and intercellular signaling. Following nerve injury, neuronal pathways involved in the regeneration of damaged axons are activated in attempts to recover lost motor, sensory, and autonomic functions. These regenerative mechanisms involve sprouting from the end of the damaged axon toward the denervated segments of the cell, collateral branching of the undamaged neurons around the injury, and long-term reorganization of neuronal circuits in the CNS in response to aberrant peripheral signals [25]. Changes in gene expression and redistribution of receptors and ion channels in the neuronal membrane [26] may partially compensate for incomplete reinnervation; however, altered sensation and loss of fine motor control may produce functional deficits as well as maladaptive alterations (e.g., enhanced spinal reflexes, neuropathic pain).

The few existing studies of sex differences in nerve regeneration suggest that regeneration of myelinated neurons occurs more rapidly in females than males [27–29]. However, Tong and colleagues recently found no sex differences in axon regeneration of axotomized neurons [30]. Our findings support the latter study since after CCI no significant sex-specific transcriptional differences exist in genes known to be involved in axon regeneration and neuron projection development. Discrepancies in neuron regeneration timing may be attributed to differences of nerve injury models, measures of regeneration, and timing of assessments.

### Gene expression profiles after injury differ between males and females

After nerve injury, male and female rats showed transcriptional changes in genes that share common GO biological processes (i.e., Behavior, Regulation of ion transport). However, less than one fourth of the genes that were enriched for each of these common processes are shared between males and females. For example, *Akap9* (A-kinase anchor protein 9) is a member of the Regulation of Ion Transport GO biologic process and, in our study, is significantly upregulated in females but not in males after CCI. *Akap9* interacts with members in signaling transduction pathways (e.g., protein kinase A, serine/threonine kinase protein kinase N, protein phosphatase 1) [31] and loss-of-function mutations are associated with Long QT syndromes [32]. The role of *Akap9* in peripheral nerves has not been reported; however, its link to regulating membrane potential suggests *Akap9* could contribute to a sex-specific pain mechanism by increasing neuron excitability after nerve injury in females.

*Oprm1* is also represented in the Regulation of Ion Transport GO biologic process, but in contrast to *Akap9*, *Oprm1* is significantly upregulated after injury in males but not in females. Importantly, *Oprm1* encodes the μ-opioid receptor (MOR) which is targeted by the agonist morphine to produce profound analgesia. Zubieta and colleagues [33] found that, in relation to women, men experienced increased activation of the endogenous analgesic mechanisms in several brain regions during infusion of hypertonic saline into the masseter muscle as demonstrated by increased binding of radiolabeled carfentanil to MORs. Further, several preclinical studies report that morphine produces greater analgesia in male animals in the setting of chronic inflammatory pain [34, 35]. In the present DRG-focused study, the higher *Oprm1* expression in males after injury unveiled a possible peripheral mechanism that contributes to sex differences in morphine analgesia and endogenous analgesic mechanisms. Consistent with this finding, we previously demonstrated a relative decrease in efficacy of a peripherally-acting MOR agonist DALDA (dermorphin [D-Arg2, Lys4 (1–4) amide) to inhibit neuropathic pain-related behavior after systemic administration in female rats versus male rats [36].

Another important DRG transcript we found upregulated after CCI in both male and female rats was *Csf1* (colony-stimulating factor 1). *Csf1* is transported from the DRG to the spinal cord dorsal horn where it binds to its receptor (i.e., *Csf1r*) located on microglia whose activation promotes the CNS changes necessary for mechanical hypersensitivity [37]. Our results are consistent with a recent study [37] in which *Csf1* was reported to be expressed de novo in injured sensory neurons following peripheral nerve injury. Importantly, we found *Csf1* expression was 1.7 times higher in females than in males. Further studies will be required to examine if and how sex-specific regulation of *Csf1* factors into differences between males and females in their susceptibility to chronic pain syndromes.

**Fig. 7:**
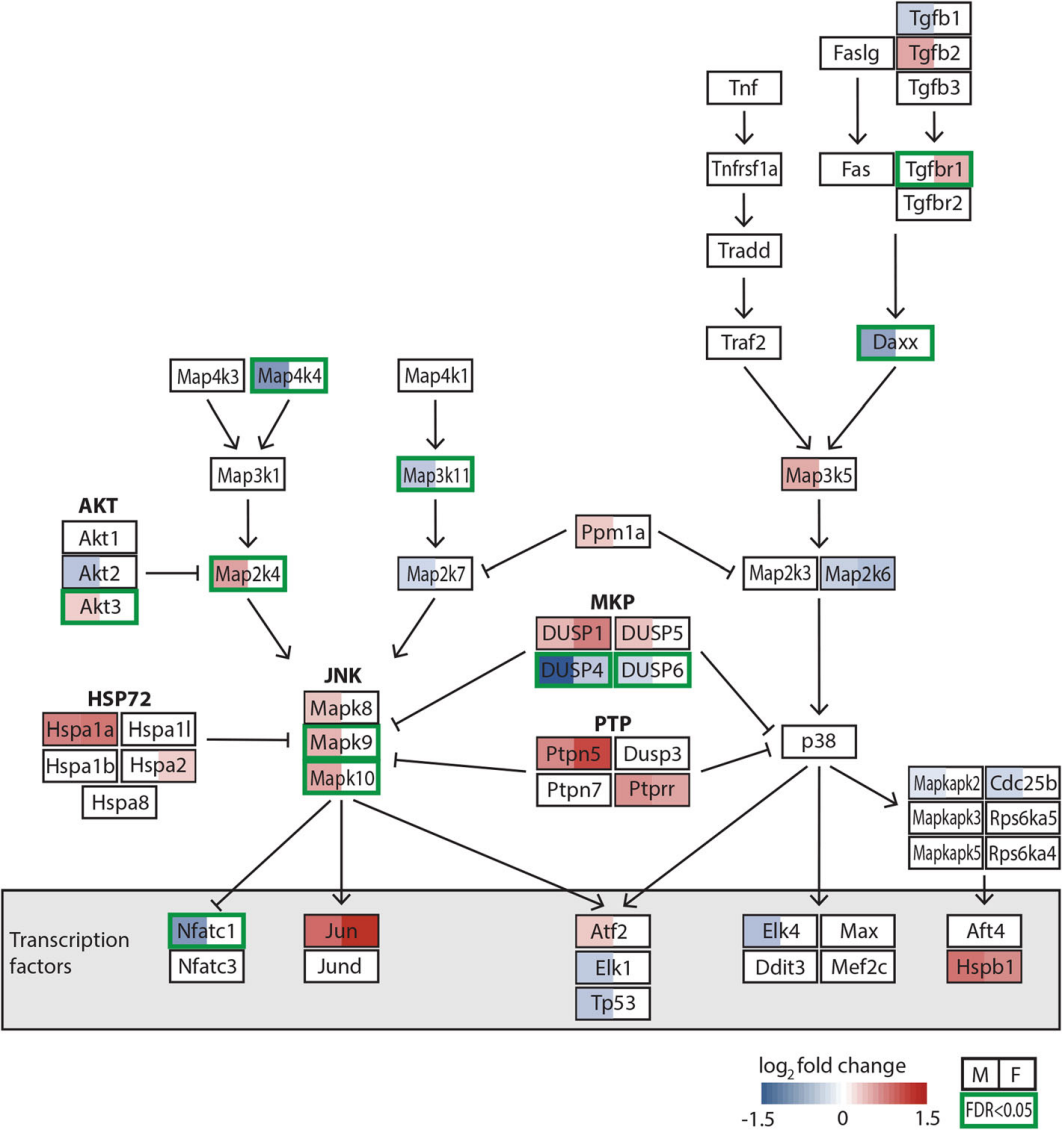
Modified KEGG map of the JNK and p38 MAP kinase signaling pathway showing transcriptional changes. Rectangles represent key genes within the pathway. Coloration in the left half and right half of each rectangle indicates the log_2_FC in gene expression following CCI versus naïve for male (left) and female (right). Only gene expression changes with an FDR < 0.05 are shown. Red shades indicated increased gene expression after CCI compared with naïve and blue shades indicated decreased gene expression as shown on the color key. Green frames identify genes that show differential gene expression between female and male DRGs with an FDR < 0.05. *DRG* dorsal root ganglion, *CCI* chronic constriction injury, *FDR* false discovery rate, *FC* fold change

We also identify many growth factors, hormones, cytokines, and neurotransmitters that are differentially up- and downregulated following CCI in males and/or females. Once these molecules bind their cognate receptors on cell membranes, they induce kinase and phosphatase activity in signal transduction cascades which can ultimately modify the expression of specific transcription factors and their respective target genes. Indeed, many of the sex-specific expression differences we identified were in genes that produce key proteins in the mitogen-activated protein kinase (MAPK) pathway (Fig. 7). Alterations of the MAPK pathways are associated with the development of pain hypersensitivity (for review see [38, 39]). Prior studies have attributed MAPK signaling within DRG cells to pain phenotypes ([40–42]). However, sex-based differences were not considered in these studies. For example, a study that examined CCI in male rats found that *Jun* (c-Jun, V-Jun avian sarcoma virus 17 oncogene homolog) is upregulated in the DRG after CCI and associated with increased expression of proteins that contribute to mechanisms involved in neuropathic pain (e.g., neuropeptide Y, vasoactive intestinal peptide) [43]. While our results are consistent with this study, we found that *Jun* expression was 1.4 times greater in females than males. We also found that expression of *Fos* (FBJ murine osteosarcoma viral oncogene homolog) was also significantly upregulated in females after CCI, but downregulated in males. Since c-Jun and c-fos combine to form the AP-1 early response transcription factor, sex-specific differences in the activity of AP-1 may contribute to the differential gene expression observed after CCI.

While we were unable to resolve cell type specificity of gene expression from bulk DRG, recent single cell expression studies have identified subpopulations of neuronal and non-neuronal cells within male DRG [44, 45]. Ultimately, understanding how sex-specific differences in PNS gene expression arise at a single cell level could help facilitate drugs regimens that limit off-target effects. While our study was limited to understanding differential gene expression during the maintenance phase of nerve-injury induced chronic pain, earlier time points should yield valuable insights into transcriptional changes that occur during the developmental phase. In addition, future studies are needed to examine the functional implications of differentially expressed genes between males and females in the DRG after CCI.

## Conclusions

Our findings build upon existing knowledge of differential pain sensitivity and susceptibility to chronic pain and highlight the profound differences in peripheral mechanisms between males and females after nerve injury which has not been well studied. First, baseline differences in immune function in the PNS may predispose females to developing chronic pain conditions. Second, functional pathways relevant to both sexes that become altered after injury may have important underlying differences in gene expression. Failures of preclinical treatments to translate into humans may be at least partially due to sex-specific alterations in gene expression and existing biases for exclusion of females in preclinical studies. Increased attention to sex differences in preclinical pain studies may improve the translational relevance to clinical populations.

## Methods

### Animal models

Adult male and female Sprague Dawley rats (RRID: RGD_5508397; Harlan Bioproducts for Science, Indianapolis, IN) were randomly assigned to undergo CCI surgery or no treatment (i.e., naive control). All surgical procedures were performed by the same individual to avoid variation in technique. *All animals*: Animals were allowed to acclimate for a minimum of 48 h before any experimental procedures, housed 2–3 per cage, and given access ad libitum to food and water. *CCI surgery group*: CCI surgery to the sciatic nerve was performed as previously described [46]. Under 2% isoflurane, a small incision was made at the level of the mid-thigh. The sciatic nerve was exposed by blunt dissection through the biceps femoris. The nerve trunk proximal to the distal branching point was loosely ligated with four 4–0 silk sutures placed approximately 0.5 mm apart until the epineuria was slightly compressed and minor twitching of the relevant muscles was observed. The muscle layer was closed with 4–0 silk suture and the wound closed with metal clips. On postoperative day 14, naive and CCI rats were euthanized by overdose of isoflurane and the ipsilateral L4-L6 DRGs were quickly dissected, immediately submerged in liquid nitrogen, and stored at − 80 °C until RNA extraction. The ipsilateral L4-L6 DRGs from a single rat were pooled and define each sample. Of note, we did not identify the stage of estrus in the female rats. Existing literature suggests that freely cycling rodents do not exhibit increased variability of study outcomes including pain behaviors [10, 47–50]. In addition, during the 14-day period between surgery and tissue harvest, each rat passes through each stage of the estrus cycle multiple times during which any differences due to the stage of estrus would average out. Veterinary care and animal housing is provided by Johns Hopkins University Animal Services and the Division of Comparative Medicine.

### Behavior testing

Mechanical hypersensitivity was measured using von Frey monofilaments as previously described [51]. Animals were assessed at baseline and on day 14 after CCI (Additional file 7). The animals were placed in individual plexiglass cages on top of a wire mesh and allowed to acclimate for 1 h. Tactile stimulation to the midplantar surface of each hind paw was performed using calibrated monofilaments with gradually increasing stiffness (i.e., 0.37, 0.61, 1.23, 2.0, 4.0, 5.93, 10.0, 13.5 g). Each monofilament was applied for 4–6 s in the area between the footpads on the plantar surface of the hind paw. Monofilaments with increasing force were applied until a positive response was observed (e.g., abrupt paw withdrawal, shaking, licking). When a positive response was observed, the monofilament with the next lower force was then applied. The test continued 1) for 5 stimulations after a positive test was observed or 2) the upper or lower range of the von Frey monofilament set was obtained. The paw withdrawal threshold was determined as previously described. If a rat did not achieve at least a 50% reduction in baseline paw withdrawal threshold after 48 h or on day 14, then this animal was not used (*n* = 3). In addition to one animal of each sex that failed to show adequate reduction of withdrawal thresholds, one naïve animal was remove from analysis due to responses at a much lower mechanical threshold than normal.

### RNA isolation

Total RNA was extracted from pooled ipsilateral lumbar DRGs (L4–6) from one rat using the Qiagen RNeasy Mini Prep Kit (Qiagen, Valencia, CA; #74104) with on-column DNase digestion (Qiagen; #79254) according to manufacturer’s instructions. RNA concentration was measured using the Nanodrop ND-2000 Spectrophotometer (Thermo Fisher Scientific, Waltham, MA) and RNA integrity was assessed using RNA Nano Eukaryote chips in an Agilent 2100 Bioanalyzer (Agilent Technologies, Palo Alto, CA).

### Library construction and sequencing

Five hundred nanograms of total RNA per sample was used to construct sequencing libraries (*n* = 1 rat/sample and run in independent biological duplicates per group per sex). Strand-specific RNA libraries were prepared using the NEBNext Ultra Directional RNA Library Prep Kit for Illumina (New England Biolabs; # E7420S) after poly(A) selection by the NEBNext poly(A) mRNA Isolation Module (New England Biolabs; #X7490) according to manufacturer’s instructions. Samples were barcoded using the recommended NEBNext Multiplex Oligos (New England Biolabs; #E7490). Size range and quality of libraries were verified on the Agilent 2100 Bioanalyzer (Agilent Technologies; Palo Alto, CA). RNA-seq libraries were quantified by qPCR using the KAPA library quantification kit (KAPA Biosystems). Each library was normalized to 2 nM and pooled in equimolar concentrations. Paired-end sequencing was performed on an Illumina HiSeq2500 (Illumina, San Diego, CA). Two independent biological replicates of 1 rat per group per sex were run for a total of 8 libraries. Libraries were sequenced to an average depth of 47.6 million reads per sample in two batches. Samples from each batch were multiplexed and resequenced in a single HiSeq lane to evaluate for batch effects. Data was preprocessed using the identical pipeline and Pearson correlation coefficients were calculated (Additional file 8). No correction for batch effects was performed.

### Data analysis

Sequencing reads were aligned to annotated RefSeq genes in the rat reference genome (rn6) using HISAT2 [52]. Aligned reads were filtered to remove ribosomal RNA and visualized using the Integrative Genomics Viewer [53]. A gene count matrix that contained raw transcript counts for each annotated gene were generated using the *featureCounts* function of the Subread package in R [54] against the Ensemble rn6 transcriptome. We filtered this matrix by removing genes with zero counts across all samples and relied on the automatic and independent filtering used by DESeq2 to determine the most appropriate threshold for removing genes with low counts [55].

To identify genes that were differentially regulated following nerve injury, transcript counts were normalized and log_2_ transformed using the default normalization procedures in DESeq2 [55]. The differential expression analysis was first performed separately for each sex using default parameters. This analysis identified differentially expressed genes between the naive and CCI groups within males or females. The interaction of sex on differential gene expression after injury was evaluated by the interaction term included in the design matrix within DESeq2. All downstream analyses on RNA-seq data were performed on data obtained from DESeq2. An adjusted *p*-value (i.e., FDR) < 0.05 and an absolute log_2_ fold change > 0.5 were used to define differentially expressed transcripts between naive and injured animals. DESeq2 adjusts for multiple testing by implementing the procedures of Benjamini and Hochberg [55]. Genes with differential expression between groups were then included in pathway analysis to infer their functional roles and relationships.

Gene ontology analysis for enriched biological processes in each set of differentially enriched genes identified by DESeq2 was performed using Metascape [http://metascape.org] [56] with a minimum enrichment of 1.0 and a p-value cutoff of 0.05. Metascape compiles data monthly from publicly available resources (e.g. NCBI, Reactome, GO, KEGG) to provide comprehensive analysis of a list of genes. All significant differentially enriched genes identified by DESeq2 were used to construct network-based functional associations using the GeneMANIA algorithm [57] as a plug-in within Cytoscape version 3.4.0 [58]. Raw and processed sequencing data has been deposited in the NCBI GEO database under accession #GEO100122.

### Validation of RNA-seq by qPCR

Total RNA extracted from DRGs of a separate cohort of rats was used to confirm the relationship of gene expression trends between sexes in selected genes by qPCR. As previously described, first-strand cDNA synthesis from 500 ng total RNA in a 20 μl reaction was performed using random hexamer primers and the SuperScript III Reverse Transcriptase (ThermoFisher Scientific) according to manufacturer’s instructions. cDNA was diluted 1:4 with nuclease-free water and stored at − 20 °C. mRNA sequence for each gene was retrieved from NCBI. Forward and reverse primers for each gene were designed using the PrimerQuest Tool (IDT, Coralville, Iowa) to span one or more introns. Primers were obtained through Integrated DNA Technologies (IDT, Coralville, Iowa) and sequences are provided in Additional file 9.

Each 20 μl qPCR reaction consisted of 10ul 2X Power SYBR Green Master Mix (Thermo Fisher Scientific, Waltham, MA), 200 nM each forward and reverse primer, and 2 μl diluted cDNA. PCR of each target was performed using the 7900HT Fast Real-Time PCR system (Applied Biosystems, Waltham, MA) with the following thermocycling conditions: initial denaturation at 95 °C for 10 min followed by 40 cycles of 95 °C for 10 s and 60 °C for 60 s. Two biological replicates were assayed for each group and each biological replicate was run in triplicate for each target gene. Nuclease-free water was included in each plate as a no-template control.

PCR efficiencies of each primer set were determined using the slope of standard curves constructed with Cq values obtained from 5-fold serial dilutions of pooled cDNA from DRGs of each group (e.g., male naïve, female naïve, male CCI, female CCI). The efficiency was calculated using they formula: E = 10^–1/slope^. Dissociation curve analysis was used to identify amplification of non-specific products including primer dimers.

### Identification of an endogenous control

To identify a stable endogenous control gene for normalization of the target genes in qPCR we selected six candidate reference genes (i.e., *Sdha*, *Hmbs*, *Polr2a*, *G6pd*, *Ywhaz*, *Actb*) that showed little variation in the RNA-seq data. The expression stability of each candidate reference gene across groups was analyzed using the NormFinder function in R (https://www.moma.dk/normfinder-software) [59]. For each candidate gene, NormFinder (RRID: SCR_003387) calculates a stability value for each gene based on the genes expression variation among samples within the same group and variation between different group. This stability value enables candidate genes to be ranked according to their expression stability among different experimental conditions. The gene or pair of genes with the lowest stability value (i.e., highest expression stability) was selected as the endogenous control for relative gene expression calculations of each target gene.

### Statistical analysis

#### Differential gene expression analysis

Several pairwise comparisons were performed. First, we compared gene expression in the DRGs from naive rats in male and females to identify transcripts that are expressed at significantly higher or lower levels than in the opposite sex. Then, we identified transcripts that were differentially regulated following injury compared with the naive tissue within the same sex (i.e., male CCI versus male naive tissue, female CCI versus female naïve tissue). Last, we compared genes that were differentially regulated following injury between males and females.

#### Quantitative real-time PCR

Default settings were used to define quantification cycle (C_q_) values using SDS software version 2.4.1 (Applied Biosystems, Waltham, MA). The Cq values were averaged over three technical replicates. If the standard deviation of this average was > 0.20, the outlying replicate was removed and the Cq was averaged over the two remaining technical replicates. The 2^−ΔΔCT^ method was used to convert C_q_ values into relative gene expression for each gene [60].

## Additional files

Additional file 1: List of genes with increased expression in male versus female rats in the naive DRG. (XLSX 75 kb)

Additional file 2: List of genes with increased expression in female versus male rats in the naive DRG. (XLSX 43 kb)

Additional file 3: Overlap of differentially expressed genes after CCI between males and females in common functional pathways. (TIF 932 kb)

Additional file 4: Complete list of genes that are significantly differentially regulated between males and females. (XLSX 164 kb)

Additional file 5: Co-expression networks of differentially expressed genes. A) Schematic diagram of experiment. Male and female rats were randomly assigned to the naïve group or receive CCI. RNA-seq performed on ipsilateral L4-L6 DRGs from each animal. Differentially expressed genes (DEG) defined as genes expressed after CCI versus naïve with a |log2FC| > 0.5 and an adjusted *p*-value < 0.05. B) Log2FC expression between the CCI and naive males (x-axis) and females (y-axis) for DEGs downregulated in CCI versus naive. Threshold of |log2FC| > 0.5 (dashed lines) with an adjusted p-value< 0.05 designates DEGs in female rats only (green), male rats only (red), and in both male and female rats (brown). Venn diagram shows the numbers of DEGs identified in each of these groups. C-D) Co-expression network for differentially expressed genes in C) female rats only D) and male rats only with decreased gene expression (top) obtained from GeneMANIA. Functional pathway analysis lists the top 5 gene ontology pathways (bottom) with the FDR for each term Co-expression networks of differentially expressed genes. DRG = dorsal root ganglia; CCI = chronic constriction injury; FDR = false discovery rate. DRG = dorsal root ganglia; CCI = chronic constriction injury; FDR = false discovery rate; FC = fold change. (TIF 817 kb)

Additional file 6: qPCR validation of RNA-seq data. Quantitative PCR was used to confirm the relationship of gene expression after nerve injury of DRGs from male and female rats: A) Increased relative expression in females only was confirmed in *Ahr*, *Cdkn1a* and *Tnfaip6*, B) Increased relative expression in males only was confirmed in *Faim2*, *Phyhipl*, and *Trpm6*, C) Increased expression in both sexes after injury confirmed in *Csf1* and *Oprm1*, and D) no change in relative expression in *Sec62*, *Stk38l*. Values represent the mean standard deviation log_2_Expression of 2 biological replicates. (TIF 1039 kb)

Additional file 7: Paw withdrawal thresholds to mechanical stimulation. (TIF 218 kb)

Additional file 8: Scatterplots of the log_2_FPKM of the initially sequenced batch versus the log_2_FPKM of the resequenced batch for each sample resequenced. (TIF 506 kb)

Additional file 9: List of primers used for qPCR validation. (XLSX 13 kb)

## Abbreviations

ATP: Adenosine Triphosphate
CCI: Chronic Constriction Injury
CNS: Central Nervous System
DALDA: Dermorphin [D-Arg2, Lys4 (1–4) amide
DRG: Dorsal Root Ganglion
FC: Fold Change
FDR: False Discovery Rate
GO: Gene Ontology
IL: Interleukin
MAPK: Mitogen-activated Protein Kinase
MHC: Major Histocompatibility Complex
MOR: Morphine Opioid Receptor
NADH: Nicotinamide adenine dinucleotide
PCR: Polymerase Chain Reaction
PNS: Peripheral Nervous System
RNA: Ribonucleic Acid
