# Supplementary figures and images for: Sex differences in gene regulation in the dorsal root ganglion after nerve injury
Source: BMC Genomics. 2019 Feb 19;20:147. doi: 10.1186/s12864-019-5512-9 (PMC6381758; doi:10.1186/s12864-019-5512-9)

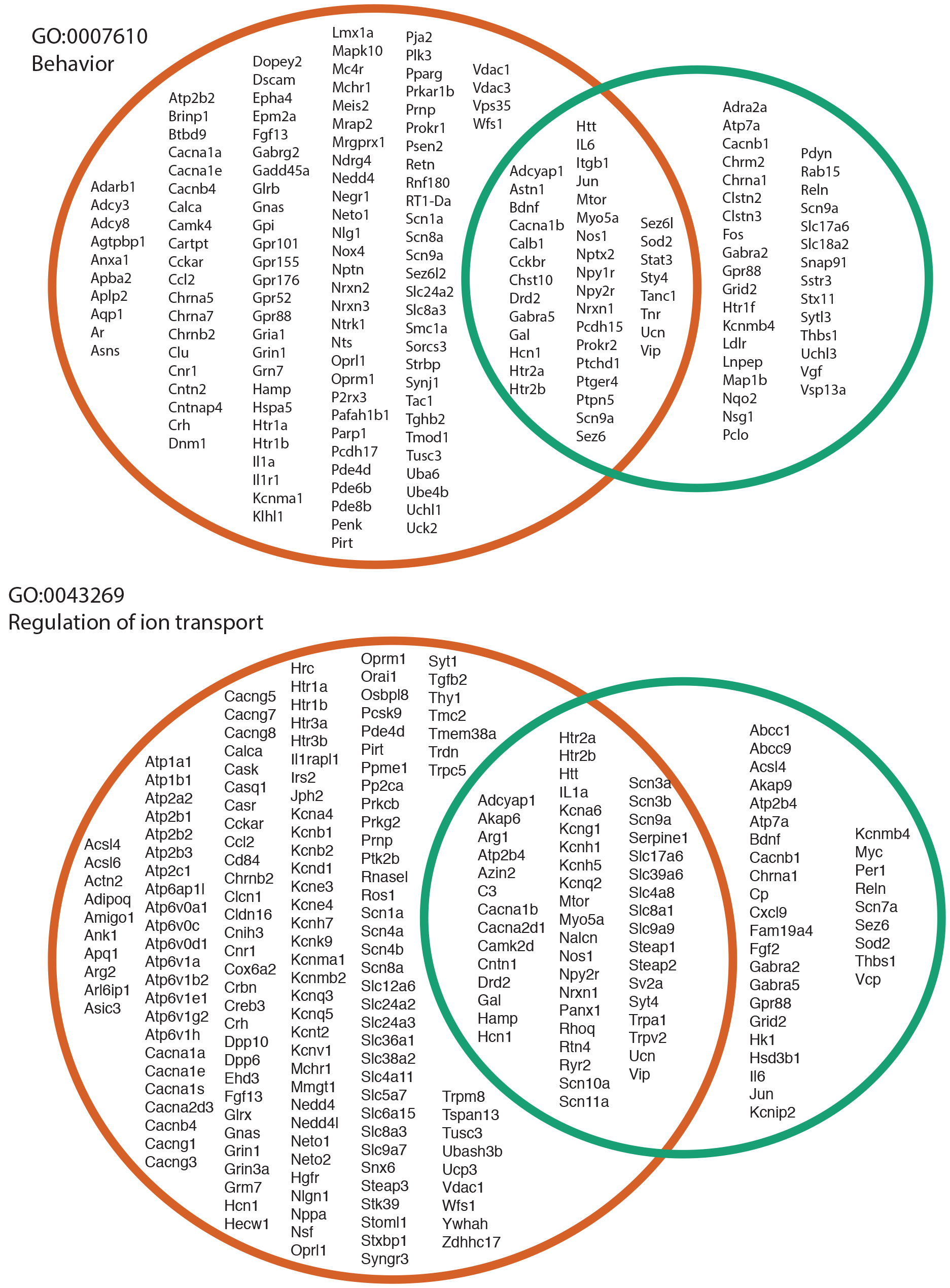

Supplement: Supplementary file 3 — Overlap of differentially expressed genes after CCI between males and females in common functional pathways. (TIF 932 kb) [file 12864_2019_5512_MOESM3_ESM.tif]

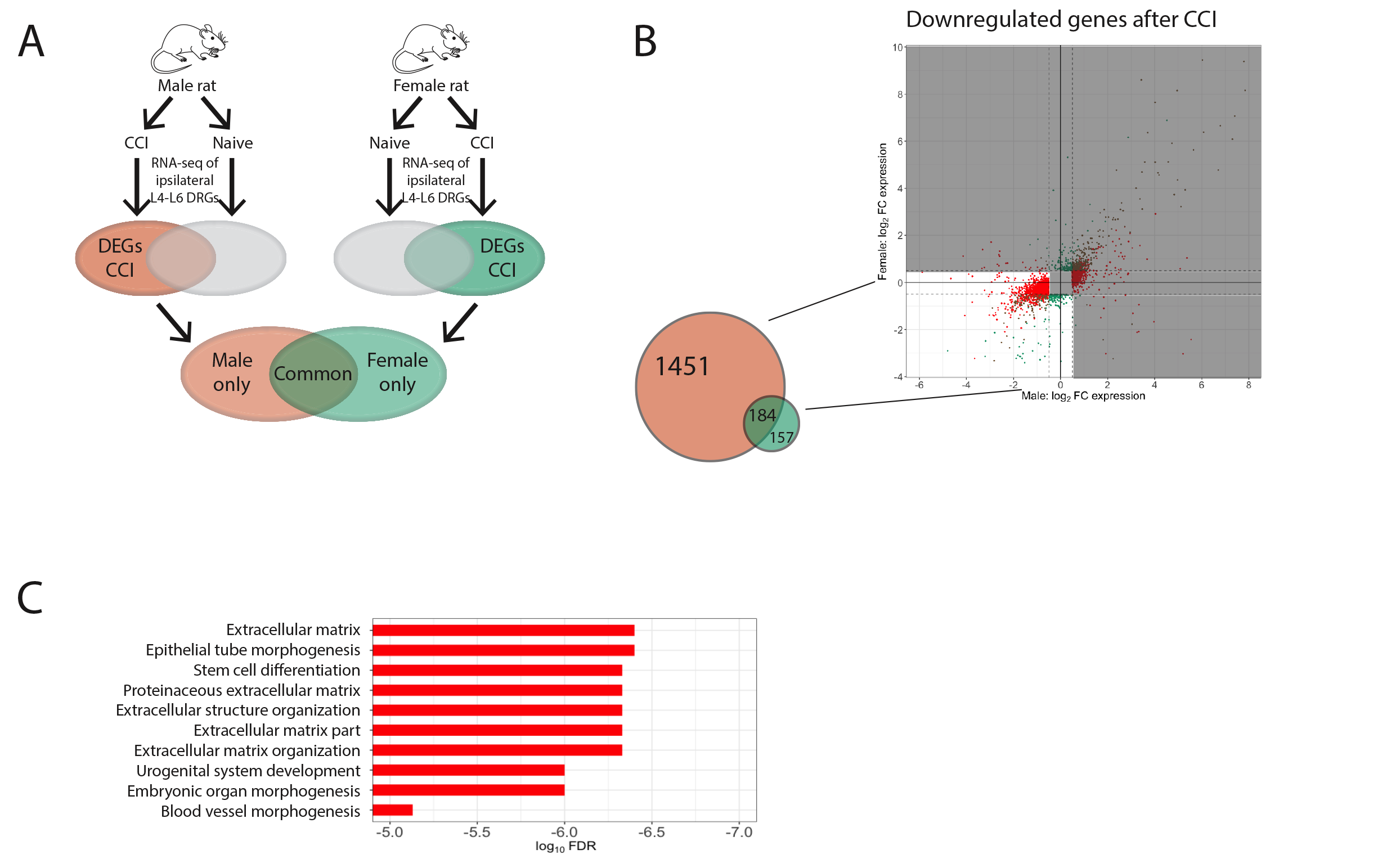

Supplement: Supplementary file 5 — Co-expression networks of differentially expressed genes. A) Schematic diagram of experiment. Male and female rats were randomly assigned to the naïve group or receive CCI. RNA-seq performed on ipsilateral L4-L6 DRGs from each animal. Differentially expressed genes (DEG) defined as genes expressed after CCI versus naïve with a |log2FC| > 0.5 and an adjusted p-value < 0.05. B) Log2FC expression between the CCI and naive males (x-axis) and females (y-axis) for DEGs downregulated in CCI versus naive. Threshold of |log2FC| > 0.5 (dashed lines) with an adjusted p-value< 0.05 designates DEGs in female rats only (green), male rats only (red), and in both male and female rats (brown). Venn diagram shows the numbers of DEGs identified in each of these groups. C-D) Co-expression network for differentially expressed genes in C) female rats only D) and male rats only with decreased gene expression (top) obtained from GeneMANIA. Functional pathway analysis lists the top 5 gene ontology pathways (bottom) with the FDR for each term Co-expression networks of differentially expressed genes. DRG = dorsal root ganglia; CCI = chronic constriction injury; FDR = false discovery rate. DRG = dorsal root ganglia; CCI = chronic constriction injury; FDR = false discovery rate; FC = fold change. (TIF 817 kb) [file 12864_2019_5512_MOESM5_ESM.tif]

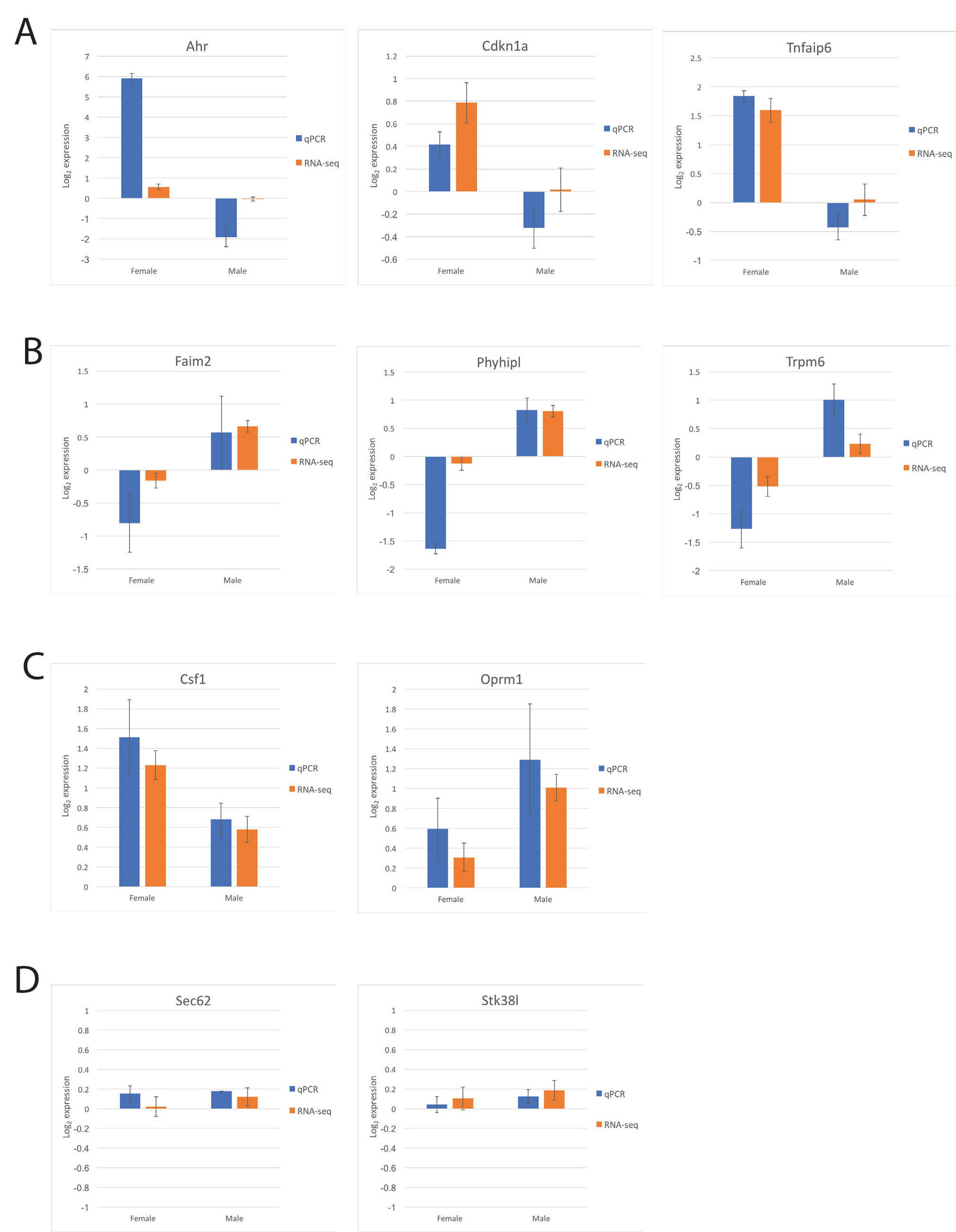

Supplement: Supplementary file 6 — qPCR validation of RNA-seq data. Quantitative PCR was used to confirm the relationship of gene expression after nerve injury of DRGs from male and female rats: A) Increased relative expression in females only was confirmed in Ahr, Cdkn1a and Tnfaip6, B) Increased relative expression in males only was confirmed in Faim2, Phyhipl, and Trpm6, C) Increased expression in both sexes after injury confirmed in Csf1 and Oprm1, and D) no change in relative expression in Sec62, Stk38l. Values represent the mean standard deviation log2Expression of 2 biological replicates. (TIF 1039 kb) [file 12864_2019_5512_MOESM6_ESM.tif]

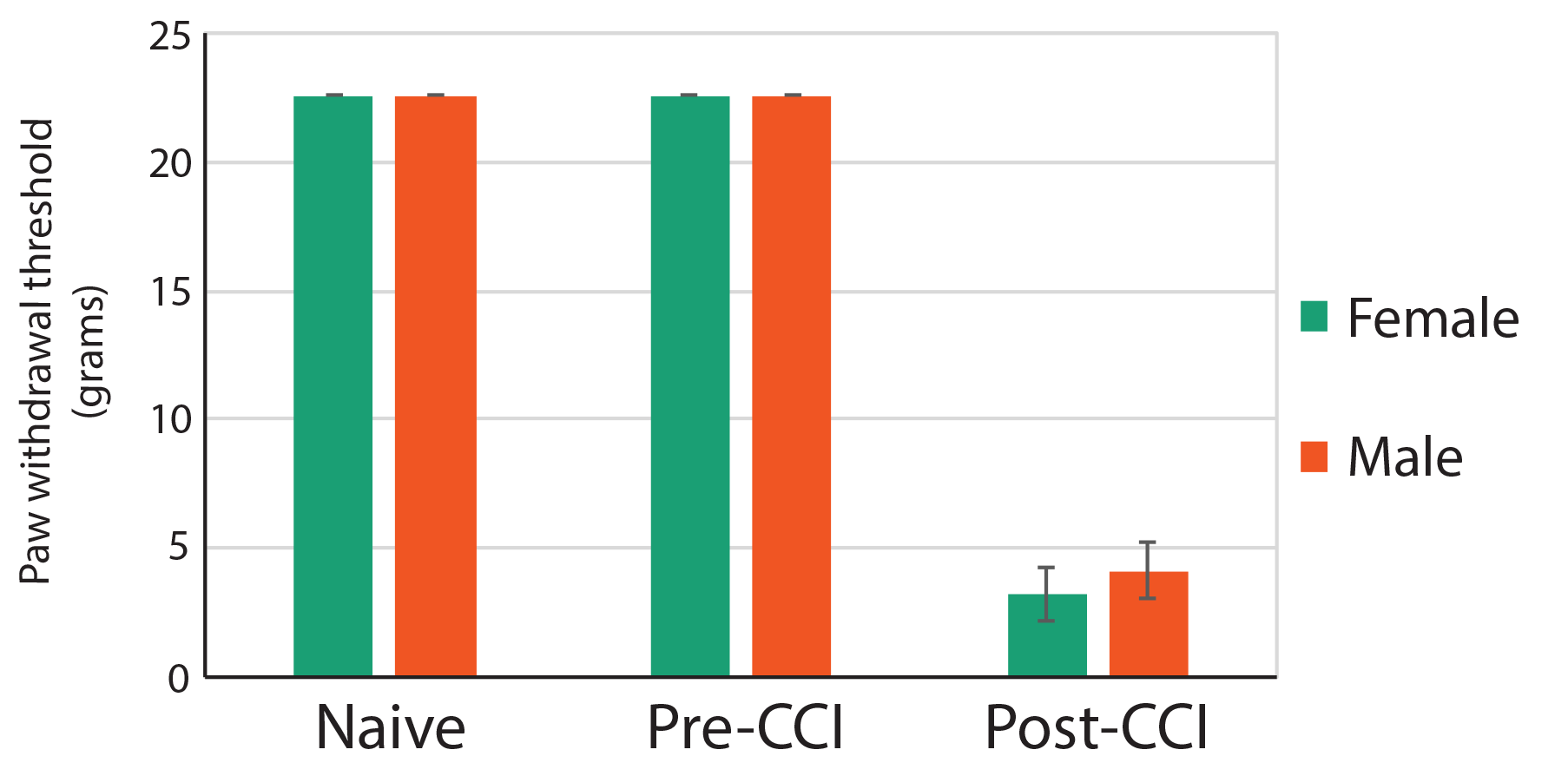

Supplement: Supplementary file 7 — Paw withdrawal thresholds to mechanical stimulation. (TIF 218 kb) [file 12864_2019_5512_MOESM7_ESM.tif]

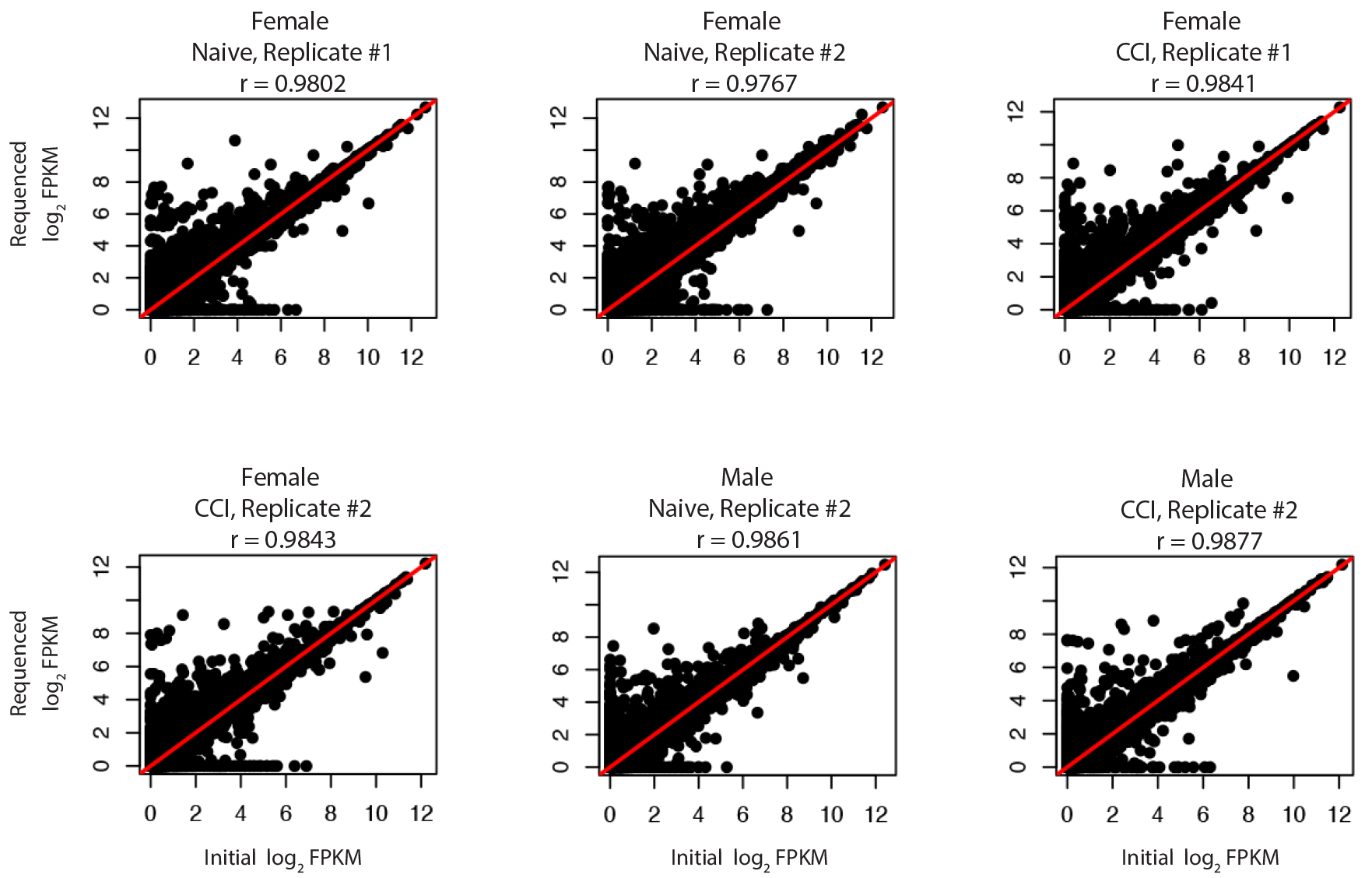

Supplement: Supplementary file 8 — Scatterplots of the log2FPKM of the initially sequenced batch versus the log2FPKM of the resequenced batch for each sample resequenced. (TIF 506 kb) [file 12864_2019_5512_MOESM8_ESM.tif]
